# TA-TMA occurring after sequential CD7 CAR-T cell therapy and allogeneic hematopoietic stem cell transplantation: a case report

**DOI:** 10.3389/fimmu.2026.1831641

**Published:** 2026-07-31

**Authors:** Jinlin Zhang, Li Ma, Xiu Liu, Yuxin An, Mohan Zhao, Mengnan Li, Xiaomei Zhang, Jile Liu, Rui Zhang, Mingfeng Zhao

**Affiliations:** 1 First Central Hospital of Tianjin Medical University, Tianjin Medical University, Tianjin, China; 2 Department of Hematology, Tianjin First Central Hospital, Tianjin, China; 3 School of Medicine, Nankai University, Tianjin, China; 4 Tianjin Thrombosis and Hemostasis Institute, Department of Hematology, Tianjin First Central Hospital, Tianjin, China

**Keywords:** allogeneic hematopoietic stem cell transplantation, CD7 CAR-T cells, eculizumab, mixed phenotype acute leukemia, transplant-associated thrombotic microangiopathy

## Abstract

Currently, CAR-T cell therapy bridging to allogeneic hematopoietic stem cell transplantation (allo-HSCT) has become a pivotal therapeutic strategy for refractory/relapsed hematologic malignancies. Sequential therapy with CD7 chimeric antigen receptor T (CAR-T) cells combined with allo-HSCT provides a novel therapeutic approach for T-lymphoid malignancies, CD7-highly expressed acute myeloid leukemia (AML), and mixed phenotype acute leukemia (MPAL). This strategy circumvents the toxicities of conventional myeloablative conditioning chemotherapy and immunosuppressive agents, while achieving tumor eradication, hematopoietic reconstitution, and prevention of graft-versus-host disease (GVHD). Transplant-associated thrombotic microangiopathy (TA-TMA) is a rare but life-threatening complication following allo-HSCT, associated with elevated non-relapse mortality (NRM). This article reports a case of early-onset TA-TMA in a patient with MPAL after CD7 CAR-T bridging to allo-HSCT. This study aims to enhance clinicians’ awareness of the diagnosis and management of TA-TMA following CAR-T bridging to allo-HSCT, provide a reference for early identification and timely intervention, and further improve patient outcomes.

## Introduction

CAR-T cell therapy has advanced rapidly in the field of hematologic malignancies, inducing remission in numerous patients with refractory or relapsed leukemia (1). In recent years, CD7 CAR-T cells have demonstrated remarkable therapeutic efficacy in patients with T-lymphoid hematologic malignancies, as well as in selected patients with CD7-highly expressed AML and mixed phenotype leukemia, with 87% of patients achieving complete remission (CR) (2, 3). Clinical evidence indicates that the efficacy of single-agent CAR-T cell therapy is not durable; therefore, rapid bridging to allo-HSCT is essential to sustain remission (2, 4, 5). The integrated strategy of CD7 CAR-T cell sequential therapy followed by allo-HSCT, developed by investigators, avoids conventional myeloablative conditioning chemotherapy and immunosuppressive agents, while concurrently achieving tumor clearance, hematopoietic reconstitution, and GVHD prophylaxis, thus opening a new avenue for allo-HSCT (6).

Clinically, complications of allo-HSCT are key factors limiting long-term survival. TA-TMA is an endothelial dysfunction syndrome occurring after allo-HSCT, characterized by microangiopathic hemolytic anemia (MAHA) with persistent schistocytosis, elevated hemolytic markers, thrombocytopenia, and microvascular thrombosis leading to ischemic injury of the kidneys and other organs (7, 8). TA-TMA is a rare but extremely severe complication with high mortality. Thus, early diagnosis and timely treatment are critical in clinical practice.

This article reports a clinical case of TA-TMA in a patient diagnosed with MPAL after receiving integrated therapy with CD7 CAR-T cell bridging to allo-HSCT.

## Case presentation

The patient was a 58-year-old female diagnosed with MPAL accompanied by myeloid sarcoma in November 2023. At initial diagnosis, bone marrow flow cytometry identified two abnormal blast populations: abnormal T-lymphoblasts accounting for 37.51% of nucleated cells and abnormal myeloid blasts accounting for 12.38%. Chromosomal analysis revealed a clonal abnormality: del(7p). Fusion gene testing was negative. Gene mutations, including CSF3R, DNMT3A, IKZF1, and NOTCH1, were detected. The final diagnosis was MPAL (myeloid/T-lymphoid [M/T] type).

**Figure 1:**
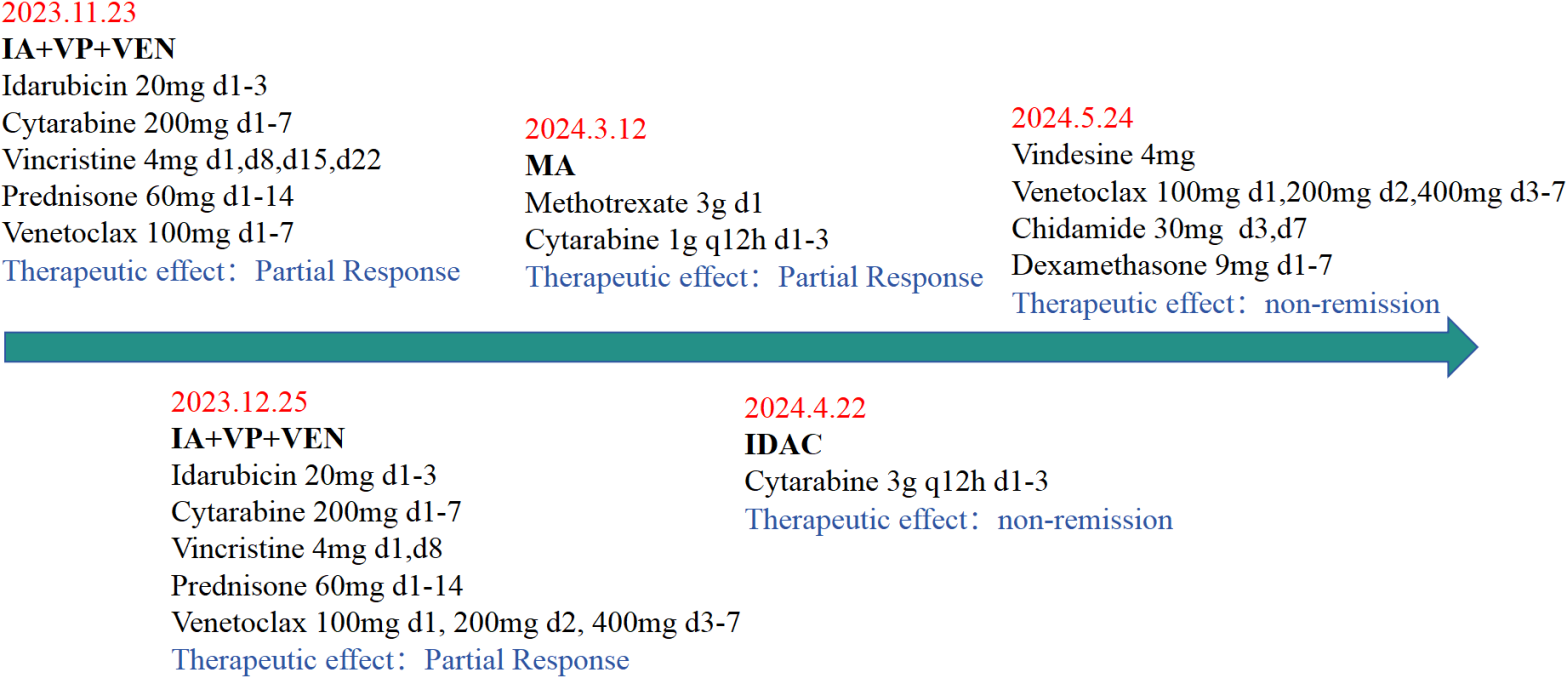
Timeline of previous treatments and efficacy evaluations of the patient.

After multiple cycles of chemotherapy with poor response, the patient was admitted to our hospital on May 29, 2024, for further treatment. The patient’s prior treatment course is illustrated in **Figure 1**. Bone marrow examination was performed on admission. Bone marrow morphology showed lymphoblasts accounting for 28% of nucleated cells with occasional monoblasts, indicating a non-remission status after treatment for acute mixed lineage leukemia, consistent with refractory acute leukemia. Bone marrow flow cytometry revealed that 15.98% of nucleated cells exhibited an immunophenotype of bright CD7, CD34+, CD1a+, weak cytoplasmic CD3, CD99+, cytoplasmic TdT+, and CD33+, consistent with abnormal T-lymphoblasts. After obtaining informed consent from the patient and her family, the patient was enrolled in a clinical trial and received CD7 CAR-T cell therapy bridging to allo-HSCT (ChiCTR2000041054).

**Figure 2:**
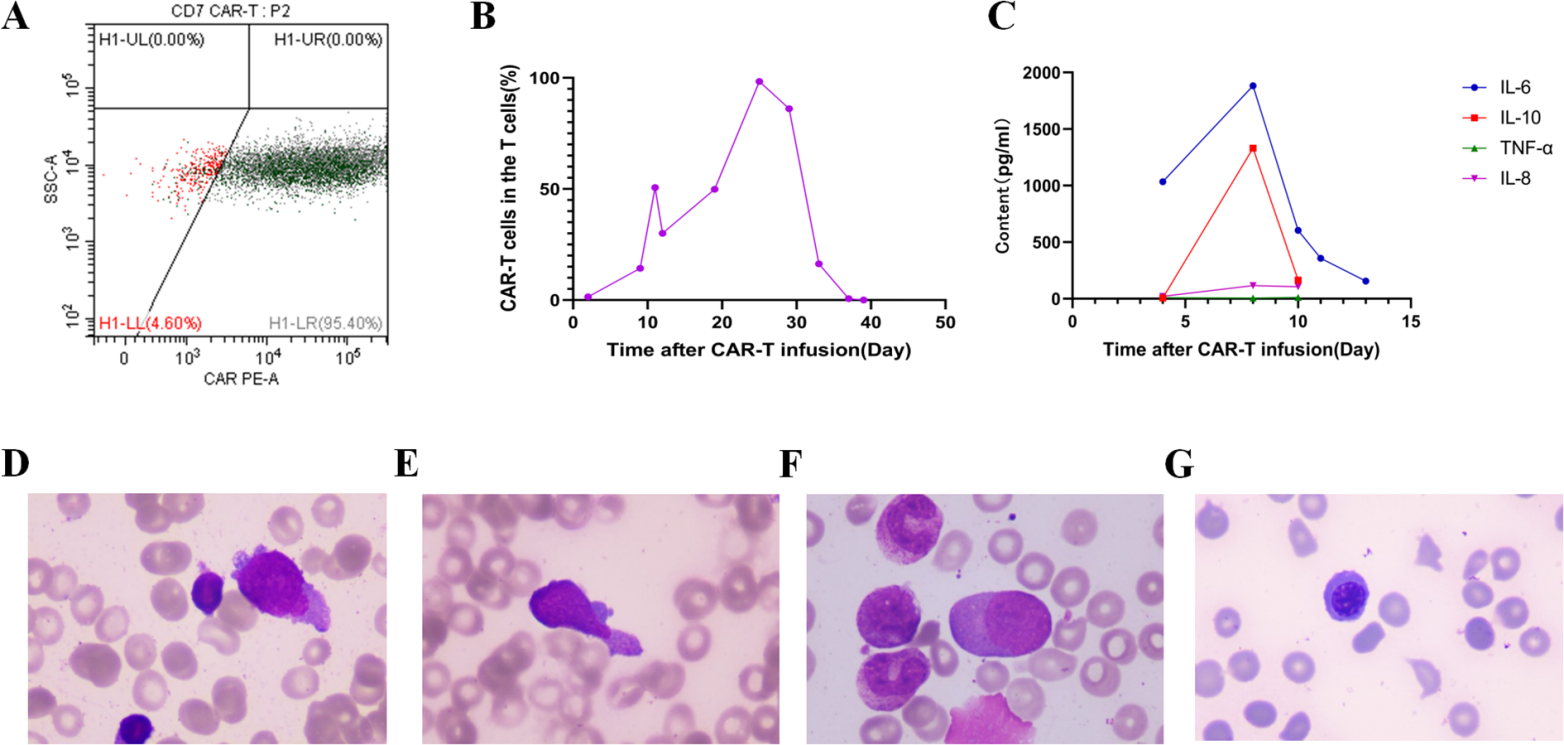
Clinical data monitoring during CAR-T cell infusion. **(A)** Flow cytometry plot of CAR-T cell transduction efficiency; **(B)** Expansion of CAR-T cells in peripheral blood; **(C)** Assessment of cytokine profiles (IL-6, IL-10, TNF-α, IL-8) during CAR-T cell therapy and allo-HSCT; **(D-F)** Bone marrow morphology before and after CD7 CAR-T cell infusion; **(G)** Morphology of peripheral blood schistocytes.

The CD7 CAR-T cells used in this case were second-generation CAR-T cells. The plasmid construct contained a CD7-targeting single-chain variable fragment (scFv), human 4-1BB and CD3ζ signaling domains, and was integrated and packaged into the pCDH-MSCV-MCS-EF1-T2A-Puro vector. Endogenous CD7 expression on CAR-T cells was downregulated by a CD7 protein expression blocker (PEBL) to prevent the “fratricide” effect among CAR-T cells (9, 10). T cells were derived from a haploidentical donor. Following T-cell isolation, activation, lentiviral transduction, expansion culture, transduction efficiency detection, quality control, cell infusion, and post-infusion monitoring, the transduction efficiency was determined to be 95.40% (**Figure 2A**).

Before CD7 CAR-T cell infusion, the patient received 5 days of lymphodepleting chemotherapy consisting of fludarabine 50 mg, cyclophosphamide 50 mg, and etoposide 100 mg daily. Prior to CD7 CAR-T infusion (June 5, 2024), bone marrow flow cytometry demonstrated that the abnormal cell population accounted for 15.98% of nucleated cells, with an immunophenotype of strongly positive CD7, CD34+, CD1a+, weak cytoplasmic CD3, CD99+, cytoplasmic TdT+, and CD33+, consistent with the abnormal T-lymphoblast immunophenotype (**Figure 2D**). A total of 4×10^6^/kg donor-derived CD7 CAR-T cells were infused on June 9 and 10, 2024.

After infusion, CAR-T cell expansion was closely monitored. The expansion of CAR-T cells during treatment was assessed, and the peak proportion of CAR-T cells in peripheral blood CD3+ cells was observed on day 25 (**Figure 2B**). Following CAR-T cell infusion, the patient developed fever with a peak temperature of 39°C. Cytokine monitoring revealed a marked elevation of interleukin-6 (IL-6), peaking at 1881.93 pg/mL (**Figure 2C**). Clinical manifestations were consistent with grade 2 cytokine release syndrome (CRS). The patient received glucocorticoids, tocilizumab combined with ruxolitinib to suppress the inflammatory response, and CRS symptoms resolved. No neurotoxicity was observed from CAR-T cell infusion to bone marrow evaluation. Bone marrow assessments were performed on days 10 and 14 after CD7 CAR-T cell infusion. On day 10 after CD7 CAR-T infusion (June 19, 2024), bone marrow flow cytometry showed disappearance of the CD7-positive abnormal blast population, with the proportions of myeloid lineage cells at all stages returning to normal. On day 14 after CD7 CAR-T infusion (June 23, 2024), bone marrow flow cytometry reconfirmed the absence of the CD7-positive abnormal cell population, and the proportions of CD34-positive progenitor cells and CD3-positive T cells were within normal ranges, confirming that the patient achieved morphological and flow cytometric complete remission (CR) (**Figures 2D–F**).

Subsequently, the patient underwent haploidentical allo-HSCT using the same donor. Per the recent clinical study protocol, no myeloablative chemotherapy or GVHD prophylaxis was administered prior to stem cell infusion (5). Before allogeneic hematopoietic stem cell infusion (June 25, 2024), the patient’s complete blood count results were as follows: white blood cell count (WBC) 1.77×10^9^/L, hemoglobin (HGB) 68 g/L, and platelet count (PLT) 13×10^9^/L. The patient was not in severe pancytopenia, vital signs were stable, and the criteria for stem cell infusion were met. On June 26, 2024, the patient received peripheral blood stem cells from the donor (daughter to mother, A+ to O+). The infused mononuclear cell dose was 7.51×10^8^/kg, and the CD34-positive cell dose was 4.824×10^6^/kg. The infusion process was uneventful. Notably, per the integrated CD7 CAR-T bridging to HSCT protocol of our center, the patient did not receive any calcineurin inhibitor (CNI) or other conventional GVHD prophylactic agents (such as methotrexate or mycophenolate mofetil) throughout the post-transplant period. On day 11 after stem cell infusion, the patient developed altered mental status, high fever, and hypotension, and was clinically diagnosed with septic shock. Pathogen testing was performed immediately, and peripheral blood cultures were positive for Escherichia coli and Klebsiella pneumoniae. Treatment with imipenem/cilastatin combined with eravacycline to cover Gram-positive and Gram-negative bacteria, plus ceftazidime-avibactam and carrimycin to cover drug-resistant bacteria, was administered. With aggressive supportive interventions including vasopressor support and cardiac function support, the patient’s condition improved.

On day 14 post-transplant, the patient developed conjunctival hemorrhage. Blood pressure was 150/83 mmHg. Laboratory investigations revealed soluble C5b-9 (sC5b-9) 2582 ng/mL, random urine protein-to-creatinine ratio (rUPCR) 1753.86 mg/g, lactate dehydrogenase (LDH) 638.30 U/L, HGB 62 g/L, and PLT 2×10^9^/L. Red blood cell morphology showed 2% schistocytes (**Figure 2G**), indicating microangiopathic hemolysis. Based on the diagnostic criteria proposed by Jodele et al. (11), a diagnosis of TA-TMA was established.

Differential diagnoses between TA-TMA and other TMAs and coagulopathies were performed simultaneously: The classic clinical features of thrombotic thrombocytopenic purpura (TTP) are the “pentad” (thrombocytopenia, microangiopathic hemolysis, neuropsychiatric symptoms, fever, and renal insufficiency). Except for thrombocytopenia and hemolysis, this patient lacked typical TTP neuropsychiatric symptoms (such as confusion, seizures, or focal neurological deficits) and fever (fever was clearly attributable to septic shock). Furthermore, markedly elevated sC5b-9 (2582 ng/mL, >10 times the upper limit of normal) provided direct evidence of terminal complement pathway activation, which is not typically observed to this extent in classic TTP (characterized by ADAMTS13 deficiency leading to accumulation of ultra-large von Willebrand factor [vWF] multimers). Therefore, comprehensive clinical judgment confirmed TA-TMA rather than TTP. Disseminated intravascular coagulation (DIC) typically presents with prolonged prothrombin time (PT)/activated partial thromboplastin time (aPTT), decreased fibrinogen (consumptive coagulopathy), and markedly elevated D-dimer. In this patient, PT, aPTT, and fibrinogen levels were all within normal ranges, failing to meet the International Society on Thrombosis and Haemostasis (ISTH) DIC diagnostic criteria (ISTH DIC score <5). Elevated D-dimer could be attributed to microvascular thrombosis rather than consumptive coagulopathy. Although the patient developed septic shock on day 11 post-transplant, coagulation tests did not indicate DIC. Sepsis-associated coagulopathy usually presents with mildly prolonged PT/aPTT, progressive platelet decline, and elevated D-dimer; however, this patient’s normal coagulation times and normal fibrinogen were inconsistent with the typical laboratory features of sepsis-associated coagulopathy. The changes in key laboratory parameters before and after diagnosis all pointed toward the diagnosis of TA-TMA (**Supplementary Figure 1**).

TA-TMA is a severe and high-risk complication after HSCT, associated with a risk of multi-organ failure and an extremely poor prognosis. Given the patient’s concurrent hepatic and renal insufficiency, prompt plasma exchange combined with eculizumab therapy was recommended. The patient’s family consented to eculizumab but refused plasma exchange. Eculizumab was administered at a dose of 900 mg per dose on days 14, 16, 20, and 27 post-transplant, for a total of four doses. Laboratory tests showed that sC5b-9 was 375 ng/mL on day 20 post-transplant, and by day 27 post-transplant, sC5b-9 and other relevant indicators returned to normal. During this period, bone marrow morphology and flow cytometry remained negative, suggesting that the patient achieved complete remission after CD7 CAR-T bridging to allo-HSCT. Unfortunately, the patient ultimately died of cerebral infarction complicated by cerebral hemorrhage and multi-organ failure.

## Discussion

CAR-T cell therapy has evolved rapidly in the field of hematologic malignancies, inducing remission in many patients with refractory or relapsed leukemia. Currently, CAR-T cell therapy bridging to allo-HSCT has become an important therapeutic strategy for refractory/relapsed hematologic malignancies. However, conventional myeloablative conditioning and GVHD prophylaxis before allo-HSCT are associated with significant toxicities and may eliminate residual CAR-T cells, thereby impairing anti-tumor efficacy (6). In the present case, leveraging the myelosuppressive state induced by CD7 CAR-T cell therapy, allogeneic hematopoietic stem cells from the same donor were directly infused without high-dose myeloablative conditioning chemotherapy or anti-GVHD agents prior to transplantation. Based on the ability of CAR-T cells to clear CD7-positive normal T lymphocytes, GVHD can be partially prevented without immunosuppressive agents (6). This integrated protocol exhibited a favorable safety profile in the present case: no severe CRS or neurotoxicity occurred during treatment, and no severe GVHD occurred post-transplant.

Allo-HSCT is a curative treatment modality for various hematologic diseases. Despite substantial efforts before and after transplantation and some improvements in clinical outcomes, transplant-related complications remain prevalent. TA-TMA is one such severe complication; although its incidence is relatively low, it is associated with high non-relapse mortality and therefore warrants close attention (12). The pathogenesis of TA-TMA is not fully elucidated, and the “two-hit” hypothesis is currently the most widely accepted theory (13). This hypothesis proposes that the first hit occurs during the conditioning regimen and the early post-transplant period, when factors such as high-dose radiotherapy or chemotherapy and human leukocyte antigen (HLA) mismatch induce a procoagulant state in vascular endothelial cells. The second hit is triggered by post-transplant factors such as immunosuppressants (e.g., CNIs), GVHD, and infection, which cause further endothelial damage via activation of the alternative complement pathway, ultimately leading to platelet aggregation and microthrombus formation (13).

In the present case, the cytokine storm associated with CAR-T cell therapy (IL-6 peak 1881.93 pg/mL) may have replaced the first-hit role of conventional myeloablative chemotherapy, i.e., inducing initial injury and activation of vascular endothelium through high levels of inflammatory cytokines. Subsequently, septic shock occurring on day 11 post-transplant constituted the second hit, superimposing ischemia-reperfusion injury, endotoxemia, and complement activation on the already damaged endothelium, ultimately triggering the clinical manifestations of TA-TMA (diagnosed on day 14 post-transplant). This hypothesis is consistent with the chronological sequence of clinical events. However, limited by the single-case design, the opposite interpretation—that septic shock served as the first hit and CAR-T cytokines merely as a background inflammatory state—cannot be excluded. An equally plausible explanation is that both factors constituted an inseparable “dual inflammatory hit,” synergistically triggering endothelial injury and TA-TMA in the absence of myeloablative chemotherapy. Validation of these hypotheses requires more cases and mechanistic studies. The value of this report lies in proposing this hypothesis rather than confirming it.

Currently, perspectives on risk factors for TA-TMA vary across different centers. Schoettler et al. proposed that patients with any adverse prognostic features should be classified as high-risk for TA-TMA (14). These adverse features include serum C5b-9 ≥ upper limit of normal (ULN), rUPCR ≥ 1 mg/mg, clinical evidence of multi-organ dysfunction syndrome (MODS), LDH ≥ 2 × ULN, concomitant grade II–IV acute GVHD, or concomitant infection (bacterial or viral) (14). In contrast, the study by Cho et al. indicated that renal insufficiency and hepatic veno-occlusive disease (VOD) are also risk factors for TA-TMA (15). Moreover, multiple studies have explored other serum biomarkers. For example, a retrospective study by Okamura et al. demonstrated that elevated serum Ba levels on day 7 after allo-HSCT could predict poor prognosis in patients with TA-TMA (16). Additionally, Arai’s team confirmed that elevated levels of neutrophil extracellular traps (NETs) were associated with an increased risk of TA-TMA (17). Research on these serum biomarkers remains limited but merits further investigation.

In this case, TA-TMA was diagnosed on day 14 post-transplant, while septic shock occurred on day 11 post-transplant. Septic shock itself can induce thrombotic microangiopathy through the following mechanisms: (1) direct injury to vascular endothelial cells by endotoxins and inflammatory cytokines; (2) endothelial oxidative stress and dysfunction resulting from shock-reperfusion injury; (3) activation of the alternative complement pathway, promoting C5b-9 deposition; and (4) disseminated intravascular coagulation and microthrombus formation. Therefore, septic shock should be regarded as the most important potential trigger for TA-TMA in this case, with its pathogenic weight possibly exceeding that of CAR-T cytokines. Meanwhile, the contribution of CAR-T cytokines to the pathogenesis of TA-TMA cannot be completely ruled out. During the patient’s CRS, the IL-6 peak was 1881.93 pg/mL, a relatively high level. IL-6 has been shown to participate in endothelial injury by inducing vascular endothelial growth factor (VEGF), activating complement, and triggering the coagulation cascade (18). However, in this case, CRS occurred early after CAR-T infusion, temporally overlapping with the diagnosis of TA-TMA, making it impossible to completely separate the effects of the two. In addition, lymphodepleting chemotherapy (fludarabine, cyclophosphamide, etoposide) and ABO blood group incompatibility (A+ donor to O+ recipient) are also reported risk factors for TA-TMA. In summary, TA-TMA in this case was most likely caused by a combination of multiple factors, with septic shock as the primary triggering event, and CAR-T-associated cytokine storm, lymphodepleting chemotherapy, and ABO-mismatched transplantation as background risk factors. Whether CAR-T has an independent TMA-inducing effect is not currently supported by sufficient evidence and requires validation in larger studies.

In the present case, normalization of sC5b-9 levels after eculizumab administration indicated that the terminal complement pathway was effectively blocked pharmacologically. However, normalization of sC5b-9 did not halt the patient’s clinical deterioration, and the patient ultimately died of cerebral infarction complicated by cerebral hemorrhage and multi-organ failure. This coexistence of normalized laboratory parameters and fatal clinical outcome may reflect the following scenarios: (1) Existence of complement-independent pathways: in addition to complement-mediated endothelial injury, the pathological mechanisms of TA-TMA include platelet activation, microthrombus formation, and vasospasm, which are complement-independent. Eculizumab only blocks the terminal complement pathway and has no effect on these complement-independent pathological processes; (2) Superimposed effect of septic shock: the systemic inflammatory response and multi-organ hypoperfusion caused by septic shock can induce organ damage independent of the complement pathway. Even if the complement pathway is successfully blocked, infection-related organ damage may continue to progress. Therefore, sC5b-9 normalization is only a laboratory pharmacodynamic indicator and cannot be equated with clinical efficacy or organ protection. In the treatment of TA-TMA, complement blockade may be necessary but not sufficient to reverse established terminal organ damage. This observation has important implications for the early diagnosis and earlier initiation of targeted therapy for TA-TMA.

Currently, there is no uniform treatment standard for TA-TMA. Many studies have indicated that CNIs can damage vascular endothelial cells and induce TA-TMA (19); therefore, CNI discontinuation is often considered the first-line therapeutic strategy after TA-TMA onset. However, in a large-scale study by Li et al., survival of TA-TMA patients did not improve after CNI discontinuation (20). The authors attributed this to progression of GVHD following CNI withdrawal, which further aggravated TA-TMA severity (20). Thus, the decision to discontinue CNIs should be made based on the individual patient’s condition and approached with caution in patients with severe GVHD. After discontinuing CNIs, alternative GVHD-control agents, such as glucocorticoids and daclizumab, should be administered promptly and appropriately. Other therapeutic approaches, including therapeutic plasma exchange (TPE), defibrotide, eculizumab, and rituximab, have also been reported to exert beneficial effects on TA-TMA (21). In the present case, neither tacrolimus nor cyclosporine was used. The occurrence of TA-TMA in the complete absence of CNIs suggests that: (1) TA-TMA can occur independently of CNIs in the presence of other potent triggers (such as CRS and septic shock); (2) the “CNI-free” strategy of this protocol, although eliminating CNIs as a risk factor, does not completely eliminate the risk of TA-TMA, and clinicians should remain vigilant when applying this protocol.

Eculizumab is a humanized anti-C5 monoclonal antibody that prevents endothelial injury by blocking C5b-9 formation (22, 23). Monoclonal antibodies such as eculizumab have shown efficacy in complement-mediated complications post-transplant (24). In this clinical case, eculizumab monotherapy contributed to the recovery of the patient’s laboratory and clinical indicators. Nevertheless, further exploration of therapeutic strategies for TA-TMA is still required.

This case report has certain limitations. As a single case report without a control group, the generalizability of the findings is unclear and requires validation in larger studies; thus, a causal relationship between CAR-T and TA-TMA cannot be established. Furthermore, the patient was exposed to multiple risk factors simultaneously, including CRS, septic shock, and lymphodepleting chemotherapy, making it impossible to distinguish their respective independent contributions; septic shock represents the most significant competing hypothesis. In addition, incomplete antibody profiling and the absence of autopsy are also limitations of this case report.

By reporting this rare case, this study aims to demonstrate the feasibility of the sequential “integrated” treatment strategy of CD7 CAR-T cell therapy bridging to HSCT, enhance clinicians’ awareness of the diagnosis and management of TA-TMA after CAR-T cell bridging to allo-HSCT, and provide a reference for the early identification and timely intervention of such complications.

## Data Availability

The original contributions presented in the study are included in the article/**Supplementary Material**. Further inquiries can be directed to the corresponding authors.

## Supplementary material

Supplementary Figure 1 Changes in key laboratory parameters before and after the diagnosis of TA-TMA. **(A)** Assessment of PLT before and after the diagnosis of TA-TMA. **(B)** Assessment of HBG before and after the diagnosis of TA-TMA. **(C)** Assessment of WBC before and after the diagnosis of TA-TMA. **(D)** Assessment of LDH before and after the diagnosis of TA-TMA. **(E)** Assessment of rUPCR before and after the diagnosis of TA-TMA. **(F)** Assessment of ALT before and after the diagnosis of TA-TMA. **(G)** Assessment of AST before and after the diagnosis of TA-TMA.
